# A multi-mechanism chaotic evolution approach for 3D path planning of UAVs

**DOI:** 10.1038/s41598-026-55825-x

**Published:** 2026-06-05

**Authors:** Xiaoyu Cao, Yueshun He, Linlin He, Wei Lv, Hao Song, Xu Huang

**Affiliations:** 1 School of Artificial Intelligence and Information Engineering, East China University of Technology, Nanchang, 330013 Jiangxi China; 2 School of Software, East China University of Technology, Nanchang, 330013 Jiangxi China

**Keywords:** UAV path planning, Chaotic evolution optimization, Good point set, Gradient memory mechanism, Adaptive Lévy flight strategy, Engineering, Mathematics and computing

## Abstract

3D UAV path planning intends to produce safe, feasible, and optimized flight trajectories in complex 3D environments containing multiple obstacles and coupled constraints. Nevertheless, many existing optimization approaches encounter difficulties when dealing with high-dimensional constrained search spaces, since limited population diversity and premature convergence can significantly reduce optimization effectiveness. To overcome these issues, this paper presents an improved chaotic evolution optimization (ICEO) algorithm that combines several collaborative optimization strategies. Initially, a good point set initialization method is introduced to enhance the uniformity and diversity of the initial population distribution. Subsequently, a multi-scale gradient memory mechanism is developed to provide adaptive search guidance by utilizing historical evolutionary information. In addition, an adaptive Lévy flight strategy is incorporated to enhance global exploration capability while balancing exploration and exploitation performance in a more effective manner. Experimental assessments on the CEC benchmark functions show that ICEO achieves competitive convergence precision and stable optimization behavior in comparison with several representative metaheuristic algorithms. Moreover, in simulated 3D UAV path planning scenarios, the proposed approach can produce feasible, smooth, and comparatively efficient flight trajectories under multiple coupled constraints. These findings suggest that the proposed method has promising potential for constrained trajectory optimization tasks in complex 3D environments.

## Introduction

Unmanned aerial vehicles (UAVs) have demonstrated significant advantages in various flight platforms due to their high maneuverability, ease of deployment, and relatively low operating costs^1^. In recent years, this technology has rapidly matured and has been widely applied in numerous fields, including reconnaissance and surveillance^2^, agricultural pest control^3^, emergency rescue^4^, and data collection for mapping^5^. However, as the applications continue to grow and the task settings become increasingly complex, higher requirements have been placed on the autonomous flight capabilities of UAVs. These requirements mainly lie in their perception and understanding abilities in complex environments, decision-making and path planning capabilities, along with flight safety control performance.

In this situation, path planning has emerged as a core challenge for UAV autonomous navigation systems. The main goal of UAV path planning is to to generate practicable and optimized flight trajectories, while also meeting multiple constraints related to safety, flight performance, and environmental complexity^6–8^.

Compared with traditional 2D path planning, the complexity of 3D UAV path planning has significantly increased. This problem not only involves avoiding horizontal obstacles but also needs to consider terrain height, the distribution of spatial obstacles, flight kinematics constraints, and the coupling relationships among multiple environmental and dynamic factors. Therefore, 3D UAV path planning can be described as a highly nonlinear, highly constrained, and multimodal continuous optimization problem^9,10^. Such problems usually have a large search space, irregular feasible regions, and multiple local optimal solutions, which greatly increases the difficulty of achieving effective global optimization and stable convergence. Therefore, solving these problems requires an optimization algorithm that can keep diverse population distribution and balance global search with local exploitation, and achieve stable convergence under complex constraints.

In recent years, numerous methods for UAV path planning have been proposed. There are primarily two classifications for these methods: classical path planning methods and intelligent optimization methods. Classical path planning methods, such as the A* algorithm^11^, Dijkstra algorithm^12^, artificial potential field (APF) method^13^, probabilistic roadmap (PRM)^14^, and rapid exploration random tree (RRT)^15^, etc., are widely used in robot navigation due to their high computational efficiency and the ability to quickly generate feasible collision-free paths. Among them, planners based on graph search and sampling have demonstrated particularly outstanding capabilities in real-time navigation and dynamic re-planning tasks.

However, these traditional methods mainly focus on the feasibility of the path and computational efficiency, but they may encounter limitations when dealing with high-constraint continuous optimization problems involving multiple interrelated goals. In complex 3D environments, balancing the smoothness of the path, flight safety, altitude restrictions, and trajectory quality remains a challenge for many classical planning methods.

In contrast, the intelligent optimization method^16^ has gradually been applied to complex UAV trajectory optimization tasks due to its powerful global search capability and adaptability to complex problems. Among them, the metaheuristic optimization algorithm has received widespread attention because it does not require an accurate mathematical model, has strong adaptability to complex search spaces, and possesses a better capacity to avoid local optima^17,18^. Representative methods include genetic algorithm (GA)^19^, differential evolution (DE)^20^, ant colony optimization (ACO)^21^, and particle swarm optimization (PSO)^22^.

Although the above algorithms can to some extent handle path search problems in complex constraint environments, they still commonly encounter problems such as unstable initial solution quality, rapid decline in population diversity, and insufficient search ability in complex environments, which hinders the effective trade-off between global exploration and local exploitation.

Consequently, scholars have attempted to integrate chaos theory into meta-heuristic optimization structures in order to improve the overall exploration capacity of such algorithms. For example, Liu et al.^23^ added chaotic disturbances to the Particle Swarm Optimization algorithm, successfully preventing early convergence; Kumar et al.^24^ put forward the Chaotic Marine Predators Algorithm, improving the global optimization capability by means of chaotic initialization and migration strategies; Kaur^25^ integrated chaotic mapping within the Whale Optimization Algorithm for the adaptive modulation of exploration variables, enhancing the precision of the convergence process; Altay^26^ combined several different chaotic mappings into the Slime Mold Optimization algorithm, improving the population diversity and search stability; and Raj et al.^27^ proposed the Chaotic Chimp Sine Cosine Algorithm which utilizes chaotic disturbance to enhance the potential of the method to escape local minima within multimodal problems.

Overall, these studies show that chaotic mapping can improve global search performance and convergence speed to a certain extent, but most of them still suffer from low-dimensional chaos and independent evolution of individuals.

In 2025, Dong et al.^28^ proposed a CEO algorithm based on population evolution. This algorithm is based on a two-dimensional discrete memristor hyperchaotic map, using a chaotic search method for mutations, and randomly changing control parameters during crossovers to improve the diversification of the searching procedure at both the individual and population levels, achieving cooperative evolution. However, when directly applied to 3D UAV path planning, the algorithm exhibits a low proportion of feasible solutions in the initial population, lacks an adaptive mechanism for parameter control, and tends to suffer from insufficient search capability in the later stages within complex obstacle environments.

To alleviate these limitations, this paper puts forward an improved chaotic evolution optimization (ICEO) algorithm and uses it for 3D UAV path planning. In particular, The key innovations of this paper are summarized below:

1. A good point set-based initialization strategy is introduced to enhance the diversity and spatial distribution of the initial population, thereby improving the generation ability of feasible solutions and the global exploration capability within the constrained search space.
2. A direction-aware multi-scale search mechanism is designed to enhance the convergence stability and optimization effect of the solution in the later stage of optimization.
3. An adaptive Lévy flight strategy is incorporated to strengthen global exploration capability and reduce the probability of premature convergence in complex obstacle environments.

After that, the remainder of this paper is structured as follows: the section “UAV Mathematical Model” mathematically describe the 3D UAV path planning problem; “Chaotic Evolution Optimization” shows parameters, operational procedure of the original CEO algorithm; “Improved Chaotic Evolution Optimization Algorithm” details the strategy enhancements incorporated into ICEO; “Algorithm Performance Testing and Analysis” provides experimental results and comparative analyses on multiple benchmark functions; “Application of ICEO in 3D UAV Path Planning” demonstrates simulation results for UAV path planning; and “Conclusion” summarizes the findings of this study.

## UAV mathematical model

3D UAV path planning is the continuous flight track selection in complex space^29^. It is not just about reducing the length of the path, but also involves many other constraints at the same time, such as the undulation of the terrain, avoiding threats, limiting the height of the flight, and ensuring the smoothness of the flight^30^. These factors interact with each other to create strong non-linear, multi-constraint, and multi-objective features.

Within this research, the unbroken air route is divided into a certain number of control nodes, and the UAV’s overall flight path is indirectly changed by changing these important points^31^. Based on this, this work consider various factors including the length of the route, safety limits, height limitations, and the smoothness of the flight, and model the 3D UAV path planning issue as a multi-objective weighted sum optimization problem.

### Path representation and environment modeling

In 3D path planning, a reasonable representation of the path forms the foundation for modeling and optimization. Since the UAV’s flight trajectory is continuous and adjustable in space, this study employs a sequence of control points to parameterize the UAV path.

Let the UAV flight path consist of a starting point, several intermediate control points, and an endpoint, with their 3D coordinates represented as:

1 $$\:\begin{array}{c}P=\left\{\left({x}_{\mathrm{0}},{y}_{\mathrm{0}},{z}_{\mathrm{0}}\right),\left({x}_{\mathrm{1}},{y}_{\mathrm{1}},{z}_{\mathrm{1}}\right),\cdots\:,\left({x}_{n},{y}_{n},{z}_{n}\right),\left({x}_{n+1},{y}_{n+1},{z}_{n+1}\right)\right\}\end{array}$$

The starting and terminating coordinates of the UAV are defined as $\:\left({x}_{0},{y}_{0},{z}_{0}\right)$ and $\:\left({x}_{n+1},{y}_{n+1},{z}_{n+1}\right)$, $\:\left({x}_{i},{y}_{i},{z}_{i}\right)\left(i=1,\cdots\:,n\right)$ denote optimized intermediate control points.

In the real world, the UAV’s flight altitude has to consider the undulation of the land. Let the terrain height be $\:H\left(x,y\right)$, the UAV’s actual flight altitude in 3D is :

2 $$\:\begin{array}{c}{z}_{i}^{\mathrm{a}\mathrm{b}\mathrm{s}}\:=\:{z}_{i}+H\left({x}_{i},{y}_{i}\right),\:i=0,1,\cdots\:,n+1\end{array}$$

### Path cost

Path length is a metric to measure if the flights are efficient, if it will be economical on mission. On safer and feasible terms, a smaller length generally requires fewer flights. And it can use less power. Therefore, in this study, the total flight path length is taken as one of the primary optimization objectives.

Path length refers to adding up all 3D euclidean distance of two successive control point. It is defined as follows:

3 $$\:\begin{array}{c}\begin{array}{c}{F}_{\mathrm{1}}=\sum\:_{i=0}^{n}\sqrt{{\left({x}_{i+1}-{x}_{i}\right)}^{\mathrm{2}}+{\left({y}_{i+1}-{y}_{i}\right)}^{\mathrm{2}}{+\left({z}_{i+1}^{\mathrm{a}\mathrm{b}\mathrm{s}}-{z}_{i}^{\mathrm{a}\mathrm{b}\mathrm{s}}\right)}^{\mathrm{2}}}\end{array}\end{array}$$

### Safety cost

In complex combat and surveillance scenarios, there exist high-risk threat areas including radar detection zones and air defense fire zones. If the UAV’s flight path gets close to these danger sources, it could get found or hit by them quickly, which might make the job fail. Therefore, the safety of the path should be considered a key restriction so that the UAV can finish its task safely and reliably in risky surroundings^32^.

Let the set of threat regions be defined as:

4 $$\:\begin{array}{c}{T}_{k}=\left({x}_{k},{y}_{k},{r}_{k}\right),\:\:k=1,2,\cdots\:,m\end{array}$$

**Fig. 1:**
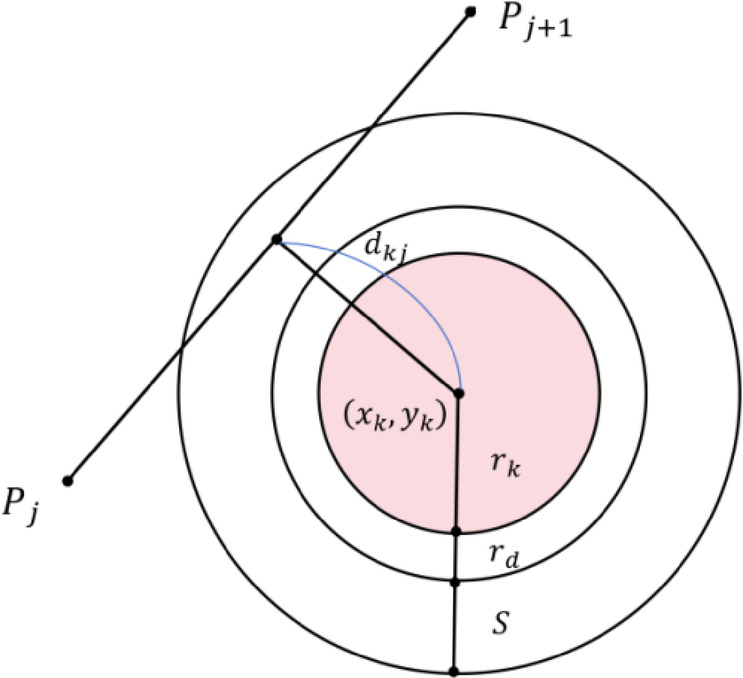
Safety optimization constraints.

Here, $\:\left({x}_{k},{y}_{k}\right)$ denote the center coordinates of the k-th threat source, and $\:{r}_{k}$ represents its threat radius, as illustrated in Fig. 1. Considering the UAV’s equivalent radius $\:{r}_{d}$ and a safety buffer distance $\:\mathrm{S}$, the smallest separation between the j-th trajectory portion and the hazard core is represented by $\:{d}_{kj}$, defined as:

5 $$\:\begin{array}{c}{d}_{kj}=DistP2S\left(\left({x}_{k},{y}_{k}\right)\mathrm{,}\left({x}_{j+1},{y}_{j+1}\right)\right)\end{array}$$

Based on the spatial correlation between flight paths and threat zones, the threat cost of a single path segment is defined as:

6 $$\:\begin{array}{c}{cost}_{kj}=\left\{\begin{array}{ll}0,&\:{d}_{kj}>{r}_{k}+{r}_{d}+S,\\\:{F}_{\infty\:},&\:{d}_{kj}<{r}_{k}+{r}_{d},\\\:\left({r}_{k}+{r}_{d}+S\right)-{d}_{kj},&\:otherwise\end{array}\right.\end{array}$$

The total threat cost is defined as:

7 $$\:\begin{array}{c}{F}_{2}=\sum\:_{k=1}^{m}\sum\:_{j=0}^{n}{cost}_{kj}\end{array}$$

### Altitude cost

In practical flight missions, UAVs are usually subject to minimum and maximum flight altitude constraints, such as ground obstacle avoidance, high-altitude communication limitations, or airspace regulation requirements. If the path altitude exceeds the allowable range, the path is considered an infeasible solution.

**Fig. 2:**
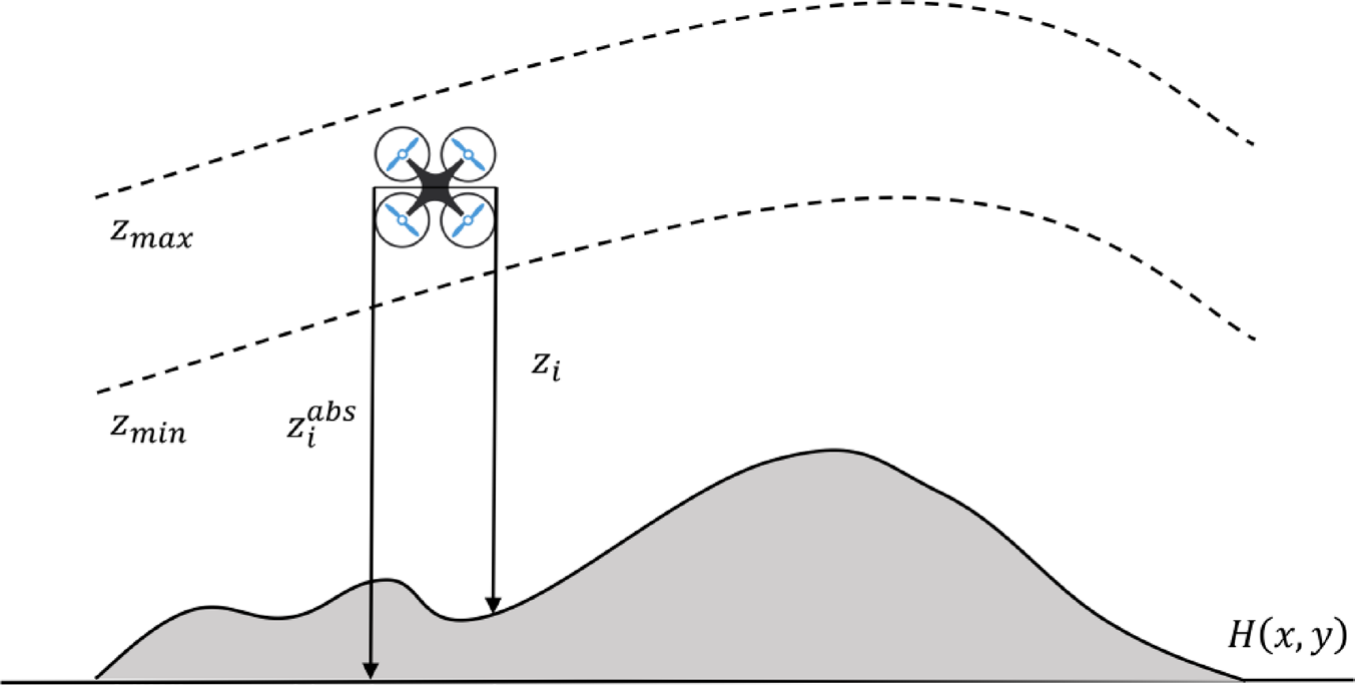
Flight altitude constraints.

Let the allowable flight altitude range be $\:\left[{z}_{min},{z}_{max}\right]$, as shown in Fig. 2. Then, The altitude cost for each control point is defined as:

8 $$\:\begin{array}{c}{J}_{3,i}=\left\{\begin{array}{ll}{F}_{\infty\:},&\:\:{z}_{i}<{z}_{min}\:or\:{z}_{i}>{z}_{max}\\\:|{z}_{i}-\frac{{z}_{max}+{z}_{min}}{2}|,&\:otherwise\end{array}\right.\end{array}$$

The total altitude cost is defined as:

9 $$\:\begin{array}{c}{F}_{3}={\sum\:}_{i=1}^{n}{J}_{3,i}\end{array}$$

### Smoothness cost

**Fig. 3:**
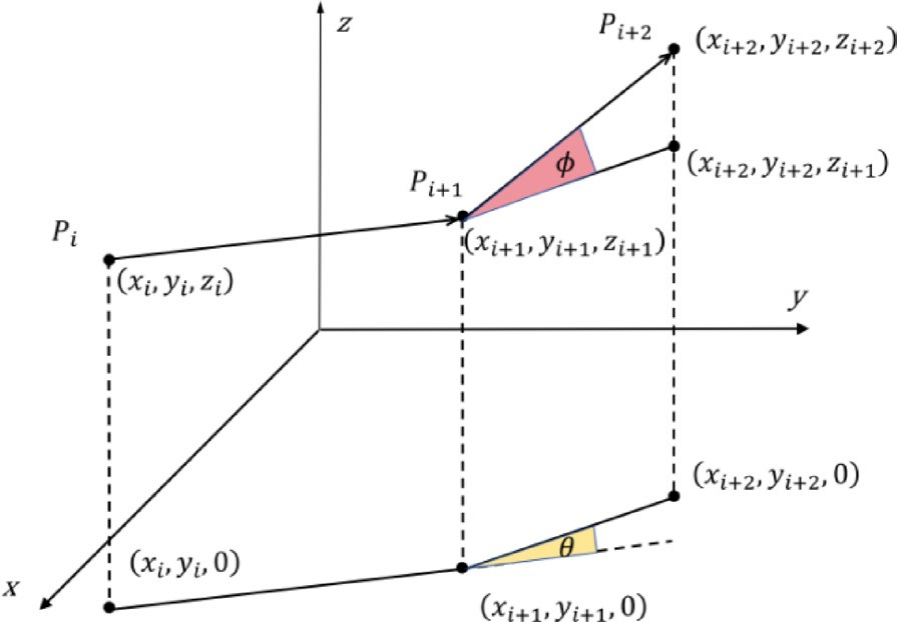
Smoothness constraints.

In addition to path length and safety, the smoothness of the flight trajectory is also a key factor affecting UAV controllability and flight stability. Excessive changes in turning angles or climbing angles can significantly increase the difficulty of flight control and may lead to violations of flight dynamic constraints^33^. From a geometric continuity standpoint, this research characterizes path smoothness with respect to the variation of turning and climbing angle between two consecutive path segments, as shown in Fig. 3.

Let the planar projection vectors of three consecutive points $\:\left(i,i+1,i+2\right)$ be defined as:

10 $$\:\begin{array}{c}{\boldsymbol{v}}_{1}=\left({x}_{i+1}-{x}_{i}\mathrm{,}{y}_{i+1}-{y}_{i},\hspace{0.17em}0\right)\mathrm{,}\text{}\mathrm{}{\boldsymbol{v}}_{2}=\left({x}_{i+2}-{x}_{i+1},\hspace{0.17em}{y}_{i+2}-{y}_{i+1},0\right)\end{array}$$

The turning angle is defined as:

11 $$\:\begin{array}{c}{\theta\:}_{i}={arctan}\mathrm{2}\left(\Vert\:{\boldsymbol{v}}_{\mathrm{1}}\times\:{\boldsymbol{v}}_{2}\Vert\:,\hspace{0.17em}{\boldsymbol{v}}_{\mathrm{1}}\cdot\:{\boldsymbol{v}}_{\mathrm{2}}\right)\end{array}$$

The corresponding climb angle is defined as:

12 $$\:\begin{array}{c}{\phi\:}_{i}={arctan}\left(\frac{{z}_{i+1}^{abs}-{z}_{i}^{abs}}{\Vert\:{\boldsymbol{v}}_{\mathrm{1}}\Vert\:}\right),\:\:{\phi\:}_{i+1}={arctan}\left(\frac{{z}_{i+2}^{abs}-{z}_{i+1}^{abs}}{\Vert\:{\boldsymbol{v}}_{\mathrm{2}}\Vert\:}\right)\end{array}$$

If $\:\left|{\theta\:}_{i}\right|>{\theta\:}_{max}$, a penalty proportional to $\:\left|{\theta\:}_{i}\right|$ is added. Similarly, if $\:|{\phi\:}_{i+1}-{\phi\:}_{i}|>{\phi\:}_{max}$, a penalty proportional to $\:|{\phi\:}_{i+1}-{\phi\:}_{i}|-{\phi\:}_{max}$ is applied.

The total smoothness cost is defined as:

13 $$\:\begin{array}{c}{F}_{4}=\sum\:_{i=0}^{n-1}\left[{max}\left(0,\left|{\theta\:}_{i}\right|\right)+{max}\left(0,|{\phi\:}_{i+1}-{\phi\:}_{i}|-{\phi\:}_{max}\right)\right]\end{array}$$

### Total cost calculation

In simple terms, 3D UAV path planning isn’t simply an optimization question for only one target, there are many criterions that needed such as route distance, safety limitation, flight altitude as well as curve. In mathematical sense this study uses the weight sum of all different sub cost to make up the entire objective function. It can be expressed as follows:

14 $$\:\begin{array}{c}Cost=\sum\:_{i=1}^{4}{b}_{i}{F}_{i}\end{array}$$

Where $\:{b}_{1}=10$,$\:{b}_{2}=100$,$\:{b}_{3}=10$ and $\:{b}_{4}=10$ are weighting coefficient for the weight that is going to be given in terms of length of path safety constraints height and smoothness. These weighting coefficients are assigned in accordance with order-of-magnitude differences between each of the individual cost terms, so that all the contributing sub-objectives add up equally into the cost function. This scaling prevents any single component from dominating the optimization process due to its larger numerical value.

## Chaotic evolution optimization

Chaotic evolution optimization (CEO) algorithm can efficiently guide the search process by integrating chaotic dynamics and evolutionary strategies organically. It leverages chaotic sequences with strong randomness and ergodicity, enabling the population to perform extensive and efficient exploration within the search space.

### Chaotic map model

The CEO algorithm leverages a 2D discrete memristive hyperchaotic map as a random number generator to boost the randomness, ergodicity, and initial-value sensitivity of the search process. Characterized by two positive Lyapunov exponents, this type of chaotic system is capable of exhibiting highly complex dynamic behaviors in phase space. Consequently, it supplies the evolutionary search with a wealth of perturbation information.

The mathematical formulation of the map is given as follows:

15 $$\:\begin{array}{c}\begin{array}{c}\left\{\begin{array}{l}{x}_{t+1}=k\cdot\:\left({e}^{-\mathrm{cos}(\pi\:{y}_{t})}-1\right)\cdot\:{x}_{t}\\\:{y}_{t+1}={y}_{t}+{x}_{t}\end{array}\right.\end{array}\end{array}$$

Where $\:{x}_{t}$ and $\:{y}_{t}$ denote the state variables of the system at the t-th iteration, respectively, and $\:\mathrm{k}$is control parameters employed to modulate the dynamic characteristics of the chaotic system. When $\:k\in\:\left(\mathrm{2.486,2.77}\right)$, the system exhibits significant hyperchaotic behavior, thereby providing high-quality chaotic sequences for the algorithm.

### Mapping individuals into chaotic space

Two individuals variables $\:{x}_{t}$ and $\:{y}_{t}$ chosen out of the existing population need to be mapped into the intervals $\:\left[-\mathrm{0.5,0.5}\right]$ and $\:\left[-\mathrm{0.25,0.25}\right]$, respectively, whereas the search space for the optimization problem is commonly set between $\:\left[lb,ub\right]$. Therefore, prior to performing chaotic search, the current individuals need to be mapped from the original solution space to the defined interval of the chaotic system to ensure the validity and consistency of the chaotic sequences.

The correlation mapping between individual state variables and chaotic variables is formulated as:

16 $$\:\begin{array}{c}\begin{array}{c}\left\{\begin{array}{l}x{{\prime\:}}_{t}=\frac{{x}_{t}-lb}{ub-lb}-0.5\\\:y{{\prime\:}}_{t}=\frac{{y}_{t}-lb}{ub-lb}\cdot\:0.5-0.25\end{array}\right.\end{array}\end{array}$$

### Generation of chaotic candidate solutions

After mapping individuals to the chaotic space, a set of chaotic candidate solutions with strong randomness and ergodicity can be generated by iterating the two-dimensional memristive hyperchaotic map. Let N chaotic candidate points be generated in each iteration, produced as follows:

17 $$\:\begin{array}{c}\left\{\begin{array}{l}{x\_chaos}^{n}=k\cdot\:\left({e}^{-\mathrm{cos}(\pi\:y{{\prime\:}}_{t})}-1\right)\cdot\:x{{\prime\:}}_{t}\\\:{y\_chaos}^{n}=y{{\prime\:}}_{t}+x{{\prime\:}}_{t}\end{array}\right.,\:n=1,2,\dots\:,N\end{array}$$

Subsequently, to allow the chaotic candidate solutions to participate in fitness evaluation and evolutionary operations, they need to be mapped back to the original search space. The inverse mapping relationship is given by:

18 $$\:\begin{array}{c}\left\{\begin{array}{l}{x\_chaos}^{n{\prime\:}}=\left({x\_chaos}^{n}+0.5\right)\cdot\:\left(ub-lb\right)+lb\\\:{y\_chaos}^{n{\prime\:}}=\left({y\_chaos}^{n}+0.25\right)\cdot\:2\cdot\:\left(ub-lb\right)+lb\end{array}\right.\end{array}$$

### Mutation operation

Mutation operation uses DE method plus a chaotic perturbation in which the difference between individuals is used as stochastic disruption and thus more different search direction are considered. Two types of mutation operators are defined. Before proceeding, it should be clarified that, without loss of generality, the following description takes the individual variable $\:{x}_{t}$ as an example, and the same procedure applies to $\:{y}_{t}$.

(1) Individual-based Mutation:

19 $$\:\begin{array}{c}{x}_{t+1}^{n}={x}_{t}+a\cdot\:\left({x\_chaos}^{n{\prime\:}}-{x}_{t}\right)\end{array}$$

(2) Best Individual-based Mutation:

20 $$\:\begin{array}{c}{x}_{t+1}^{n}={x}_{\mathrm{t}}^{*}+a\cdot\:\left({x\_chaos}^{n{\prime\:}}-{x}_{t}\right)\end{array}$$

where $\:a\in\:\left[\mathrm{0,1}\right]$ is some random number to control the size of the step to take, and $\:{x}_{\mathrm{t}}^{*}$ representing the best solution of the current generation population.

### Crossover operation

CEO algorithm also adds the diversification of populations and encourages individuals to recombine their useful information by including a Binomial Crossover operator to increase population diversity and encourage crossover among individuals that contain beneficial information. Through it, crossover is performed between the mutated ones and their parents to produce new candidates. Its mathematical description is as follows:

21 $$\:\begin{array}{c}\begin{array}{c}{x}_{j,t}^{trial}=\left\{\begin{array}{ll}{x}_{j,t+1},&\:ran{d}_{j}\left(\mathrm{0,1}\right]\le\:{C}_{r}\:or\:j={j}_{rand}\\\:{x}_{j,t},&\:otherwise\end{array}\right.\end{array}\end{array}$$

Here, $\:{C}_{r}\in\:\left[\mathrm{0,1}\right]$ is the crossover control parameter, and $\:{j}_{rand}$ denotes a random dimension index.

### Selection operation

After the crossover operation is completed, the algorithm enters the selection stage. Fitness based greedy selection strategy is used to compare the current individual with its corresponding candidate solution and select the better ones to form the next generation. The specific selection rule is defined as follows:

22 $$\:\begin{array}{c}\begin{array}{c}{x}_{t+1}=\left\{\begin{array}{ll}{x}_{t}^{trial},&\:f\left({x}_{t}^{trial}\right)\le\:f\left({x}_{t}\right)\\\:{x}_{t},&\:otherwise\end{array}\right.\end{array}\end{array}$$

**Fig. 4:**
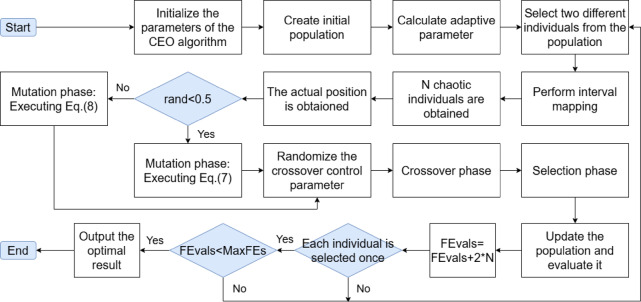
Flowchart of the CEO Algorithm.

The overall flowchart of the CEO algorithm is shown in Fig. 4.

## Improved chaotic evolution optimization

In the process of solving high dimensional complex optimization problem, the traditional chaotic evolution optimization algorithm often faces the problems of uneven distribution of the initial population, fixed parameters and insufficient search direction. To address the aforementioned drawbacks, this paper presents an Improved Chaotic Evolution Optimization (ICEO) algorithm that improves the CEO algorithm by improving its population initialization strategy, dynamic parameter control mechanism, and search strategy design.

### Population initialization according to good point set

In the original CEO algorithm, the initial population is generated using random initialization. While this strategy features simplicity and easy operability, it may result in an unbalanced population distribution in the high-dimensional search space, thereby decreasing population diversity and restricting global search capability in the initial optimization phase. The insufficient coverage of the feasible search area may also further affect the convergence stability and solution quality in subsequent iterations.

To alleviate these limitations, this paper introduces a good point set-based initialization strategy^34^. By constructing sample points with improved spatial uniformity, the proposed method enhances the coverage capability of the initial population within the search space, thereby improving feasible solution generation and global exploration performance under complex constraints^35^. The good point set theory was originally proposed by Hua Luogeng^36^, and its mathematical formulation can be expressed as follows.

Let $\:{G}_{s}$ be the unit cube in an s-dimensional space. If $\:r\in\:{G}_{s}$, then the Good Point Set $\:{P}_{n}\left(k\right)$ is:

23 $$\:\begin{array}{c}{P}_{n}\left(k\right)=\left\{\left(\left\{{r}_{1}^{\left(n\right)}*k\right\},\cdots\:,\left\{{r}_{s}^{\left(n\right)}*k\right\}\right),1\le\:k\le\:n\right\}\end{array}$$

Its discrepancy is defined as:

24 $$\:\begin{array}{c}\varphi\:\left(n\right)=C\left(r,\epsilon\:\right){n}^{-1+\epsilon\:}\end{array}$$

where $\:\left.C\left(r,\epsilon\:\right.\right)$ is a constant depending on $\:\mathrm{r}$ and an arbitrary positive number $\:\epsilon\:$.

When setting $\:r=\left\{2cos\left(2\pi\:k/\mathrm{p}\right),1\le\:k\le\:s\right\}$, where * p* is the smallest prime number that satisfies $\:\left(p-3\right)/2\ge\:s$, the obtained r is referred to as a Good Point.

In order to fit practical optimization problems, the generated Good Point Set is mapped to the search space $\:\left[lb,ub\right]$ as follows:

25 $$\:\begin{array}{c}{X}_{i,j}=\left(ub-lb\right)*r+lb\end{array}$$

**Fig. 5:**
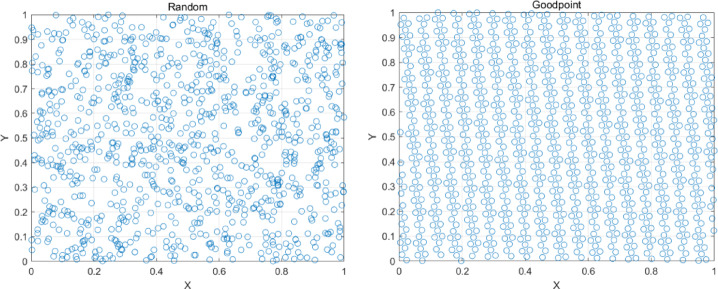
Distribution of the initial population in 2D space.

Under the conditions of a value range of [0,1], a population size of 1000, and a dimension of 2, the initial population was created using both the Good Point Set method and the random method. Distribution results are shown in Fig. 5. It can be seen from the figure that under the same number of sample points, the population distribution obtained by initializing with good point sets is much more uniform, which provides a good foundation for the algorithm’s later global search.

### Adaptive parameter dynamic control mechanism

In the CEO algorithm, key parameters such as the weight factor and exploration factor are usually fixed, making it difficult to balance the algorithm’s search requirements at different stages.

To attain a good balance of exploring globally and exploiting locally, this study designs an adaptive parameter control mechanism^37^. The basic idea is to allow key parameters of the algorithm to adjust continuously throughout the iteration process rather than remain fixed. Specifically, this study introduces the weight factor $\:{\omega\:}_{t}$ and the exploration factor $\:{\xi\:}_{t}$, whose update formulas are given as follows:

26 $$\:\begin{array}{c}{\omega\:}_{t}={r}_{\omega\:}\left(\frac{{t}^{\mathrm{2}}}{{T}^{\mathrm{2}}}-\frac{2t}{T}+\frac{\mathrm{1}}{\mathrm{2}}\right)\end{array}$$

27 $$\:\begin{array}{c}{\xi\:}_{t}={r}_{\xi\:}\left(\frac{{t}^{\mathrm{2}}}{{T}^{\mathrm{2}}}-\frac{2t}{T}+1\right)\end{array}$$

Here, $\:{r}_{\omega\:}$ and $\:{r}_{\xi\:}$ are random numbers within the range $\:\left[\mathrm{0,1}\right]$, * t* denotes the current iteration, and * T* is the maximum number of iterations. As the iterations progress, the weight factor $\:{\omega\:}_{t}$ gradually decreases, enhancing the algorithm’s local exploitation capability. At the same time, the exploration factor $\:{\xi\:}_{t}$ also reduces accordingly, enabling a smooth transition of the algorithm from coarse, large-scale search to fine, local search.

### Multi-scale gradient memory mechanism

The original CEO algorithm mainly relies on chaotic mapping and random perturbations to update the population, but it does not explicitly utilize historical search information to guide the optimization process. Therefore, during the evolutionary process, the search trajectory may experience significant fluctuations, which reduces the convergence stability and limits the refinement ability of the solution in complex constraint problems. To alleviate these limitations, the proposed ICEO algorithm introduces a direction-aware multi-scale search mechanism, providing more stable and adaptive search guidance during the optimization process.

Unlike the traditional inertia-based update strategy, which mainly relies on a single historical movement trend, the proposed mechanism simultaneously constructs short-term and long-term search memories to capture local evolutionary information and global search trends. These two memory components will adaptively fuse according to the optimization state, allowing the algorithm to sustain superior exploration capability in the initial stage and enhance convergence performance in the late iteration phase.

In addition, the direction similarity among different search memories will be evaluated to adaptively adjust the search step size according to individual circumstances, thereby enhancing the adaptability of the search under complex constraints. To further enhance the convergence stability, the fused search directions will be normalized to reduce the instability caused by scale changes, and the resulting direction vectors will be used to generate the final position update.

Take the average of the current population as the population center.

28 $$\:\begin{array}{c}{\stackrel{-}{x}}_{t}=\frac{\mathrm{1}}{N}\sum\:_{i=1}^{N}{x}_{i}\end{array}$$

To find out the possible direction for a person’s gradient according to the distance from the center of the group.

29 $$\:\begin{array}{c}{P}_{i}={\stackrel{-}{x}}_{t}-{x}_{i}\end{array}$$

30 $$\:\begin{array}{c}\:{g}_{i}={x}_{t}^{*}-C \cdot \:{P}_{i}\end{array}$$

Here, $\:{x}_{t}^{*}$ represents the position of the current best solution, and $\:C\in\:\left[\mathrm{0,1}\right]$ is a random coefficient.

To make good use of the historical search info, ICEO keeps both short-term and long-term gradient memories for every person, and the way they get updated is this:

31 $$\:\begin{array}{c}{m}_{i,short}^{\left(t\right)}={\beta\:}_{\mathrm{s}}{m}_{i,short}^{\left(t-1\right)}+\left(1-{\beta\:}_{\mathrm{s}}\right){g}_{i}\end{array}$$

32 $$\:\begin{array}{c}{m}_{i,long}^{\left(t\right)}={\beta\:}_{l}{m}_{i,long}^{\left(t-1\right)}+\left(1-{\beta\:}_{l}\right){g}_{i}\end{array}$$

Here, $\:{m}_{i,}^{\left(t-1\right)}$ represents the historical gradient memory at the previous iteration, and its initial value is set to zero. $\:{\beta\:}_{\mathrm{s}}=0.7$ controls the decay rate of short-term memory, and $\:{\beta\:}_{l}=0.95$ controls the decay rate of long-term memory. Equations (31) and (32) are using an Exponential Moving Average (EMA) to represent past gradient information. The effective memory length reflects the number of past gradient steps and can be approximately expressed as:

33 $$\:\begin{array}{c}{T}_{eff}\approx\:\frac{1}{1-\beta\:}\end{array}$$

When $\:{\beta\:}_{s}=0.7$, the corresponding effective memory length is about $\:{T}_{s}\approx\:1/\left(1-0.7\right)\approx\:3.33$. This means that the short-term gradient memory mainly shows how the search direction has changed in the last 3–4 steps, which allows the algorithm to react fast when a person changes their direction while searching locally. When $\:{\beta\:}_{l}=0.95$, the effective memory length is approximately $\:{T}_{l}\approx\:1/\left(1-0.95\right)=20$, corresponding to a long-term gradient accumulation process spanning multiple iteration cycles.

For the purpose of coordinating global search and local exploitation, the short-term and long-term gradients are combined using a weighted scheme:

34 $$\:\begin{array}{c}{m}_{i}^{\left(t\right)}={\omega\:}_{\mathrm{s}}^{\left(t\right)}{m}_{i,short}^{\left(t\right)}+{\omega\:}_{l}^{\left(t\right)}{m}_{i,long}^{\left(t\right)}\end{array}$$

The weights $\:{\omega\:}_{\mathrm{s}}^{\left(t\right)}$ and $\:{\omega\:}_{l}^{\left(t\right)}$ are dynamically adjusted according to the iteration stage:

35 $$\:\begin{array}{c}({\omega\:}_{\mathrm{s}}^{\left(t\right)},{\omega\:}_{l}^{\left(t\right)})=\left\{\begin{array}{ll}(0.7,\hspace{0.17em}0.3),&\:\frac{t}{T}<0.3\\\:(0.5,\hspace{0.17em}0.5),&\:0.3\le\:\frac{t}{T}<0.7\\\:(0.3,\hspace{0.17em}0.7),&\:otherwise\end{array}\right.\end{array}$$

Incorporating the second-moment estimation mechanism from the Adam algorithm^38^:

36 $$\:\begin{array}{c}{v}_{i}^{\left(t\right)}={\beta\:}_{\mathrm{2}}{v}_{i}^{\left(t-1\right)}+\left(1-{\beta\:}_{\mathrm{2}}\right){g}_{i}^{2}\end{array}$$

Obtain unbiased estimates through bias correction:

37 $$\:\begin{array}{c}{\widehat{m}}_{i}^{\left(t\right)}=\frac{{m}_{i}^{\left(t\right)}}{1-{\beta\:}_{\mathrm{1}}},{\widehat{v}}_{i}^{\left(t\right)}=\frac{{v}_{i}^{\left(t\right)}}{1-{\beta\:}_{\mathrm{2}}}\end{array}$$

Where $\:{\beta\:}_{1}=0.9$ is the decay rate for the first moment and $\:{\beta\:}_{2}=0.999$ for the second moment.

The corrected gradient direction is then updated as:

38 $$\:\begin{array}{c}{grad\_direction}_{i}={x}_{i}^{\left(t\right)}-{\eta\:}_{i}^{\left(t\right)} \cdot \:\frac{{\widehat{m}}_{i}^{\left(t\right)}}{\sqrt{{\widehat{v}}_{i}^{\left(t\right)}}+ϵ}\end{array}$$

Here, $\:{\eta\:}_{i}$ is the individual adaptive learning rate, and * ϵ* is a small constant to prevent division by zero. The individual learning rate is dynamically adjusted based on the consistency between the short-term and long-term gradient directions:

39 $$\:\begin{array}{c}{\eta\:}_{i}^{\left(t\mathrm{+1}\right)}=\left\{\begin{array}{ll}{max}\left({\eta\:}_{min},\hspace{0.17em}0.9{\eta\:}_{i}^{\left(t\right)}\right),&\:\mathrm{cos}{\theta\:}_{i}<0.3\\\:{\eta\:}_{i}^{\left(t\right)},&\:0.3\le\:\mathrm{cos}{\theta\:}_{i}\le\:0.8\\\:min\left({\eta\:}_{max},1.1{\eta\:}_{i}^{\left(t\right)}\right),&\:\mathrm{cos}{\theta\:}_{i}>0.8\end{array}\right.\end{array}$$

The direction consistency $\:cos{\theta\:}_{i}$ is defined as:

40 $$cos\theta _{i} = \frac{{m_{{short}} \cdot m_{{long}} }}{{\left\| {m_{{short}} } \right\|\left\| {m_{{long}} } \right\|}}$$

The final position update equation is:

41 $$\:\begin{array}{c}{x}_{i}^{\left(t\mathrm{+}\mathrm{1}\right)}={\omega\:}_{t} \cdot \:{x}_{i}^{\left(t\right)}+\left(1-{\omega\:}_{t}\right) \cdot \:{grad\_direction}_{i}+r \cdot \:\left({x\_chaos}^{n{\prime\:}}-{x}_{i}^{\left(t\right)}\right)\end{array}$$

### Global search enhancement mechanism based on Lévy flight

Original CEO algorithm mainly depends on chaotic disturbance to get the new solutions so it could get good near-by solution searching power but it does not have big far jump ability and easy to fall into local optimum. Increase global search ability by adding a Lévy flight mechanism where the flight trigger probability is updated using a Sigmoid function:

42 $$\:\begin{array}{c}{P}_{levy}\left(t\right)=\frac{\mathrm{1}}{1+exp\left(-\left(18 \cdot \:\frac{t}{T}-12\right)\right)}\end{array}$$

The Lévy step length follows a stable distribution, approximated as:

43 $$\:\begin{array}{c}{\sigma\:}_{u}={\left(\frac{\varGamma\:\left(1+\rho\:\right) \cdot \:sin\left(\pi\: \cdot \:\rho\:/2\right)}{\varGamma\:\left(1+\rho\:\right)/2 \cdot \:\rho\: \cdot \:{\mathrm{2}}^{\left(\rho\:-1\right)/2}}\right)}^{1/\rho\:},\:\:s=\frac{u \cdot \:{\sigma\:}_{u}}{{\left|v\right|}^{1/\rho\:}}\end{array}$$

Here, $\:\rho\:=1.5$ is the stability parameter, u and v are random variables that follow the standard normal distribution.

The spatial position of each individual is then iterated via the formula below:

44 $$\:\begin{array}{c}{x}_{new}={x}_{\mathrm{t}}^{*}+s \cdot \:{\xi\:}_{t} \cdot \:\left({x}_{\mathrm{t}}^{*}-\frac{2t}{T}{x}_{i}\right)\end{array}$$

**Fig. 6:**
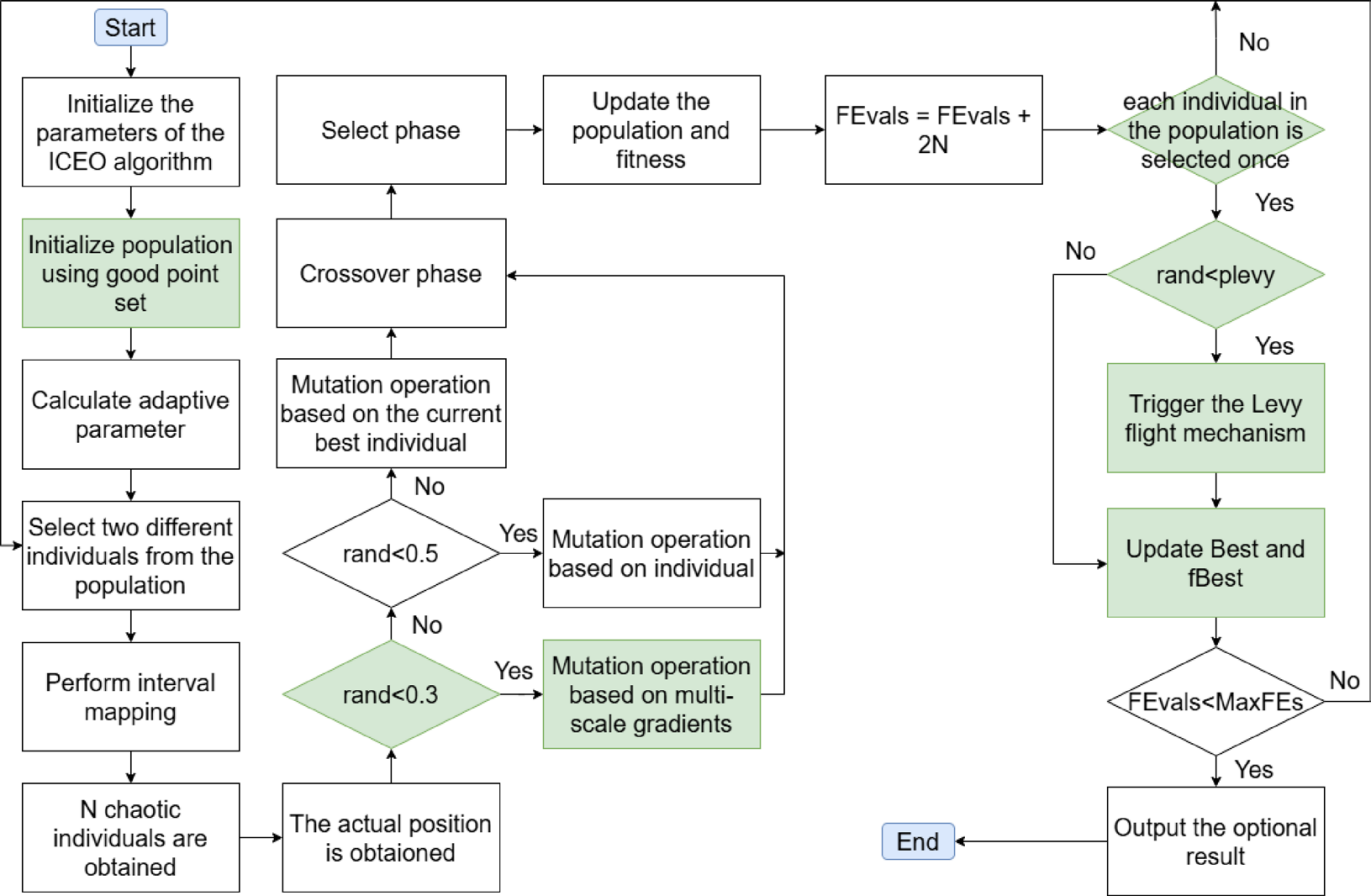
Flowchart of the ICEO algorithm.

As summarized above from Good points set initialized, Parameter adjustments, multiple scale, gradients with memories Search for, along with adding Lévy Flights in our new ICEO improved method. This combination increases both the general and local search ability of the algorithm to an optimal degree and also gives us good results in the convergence aspect as well as the solutions are fairly stable across multiple optimizations. In short it can be seen from Fig. 6 and Algorithm 1 that is showing the overall flowchart and pseudo-code of ICEO.

**Algorithm 1:**
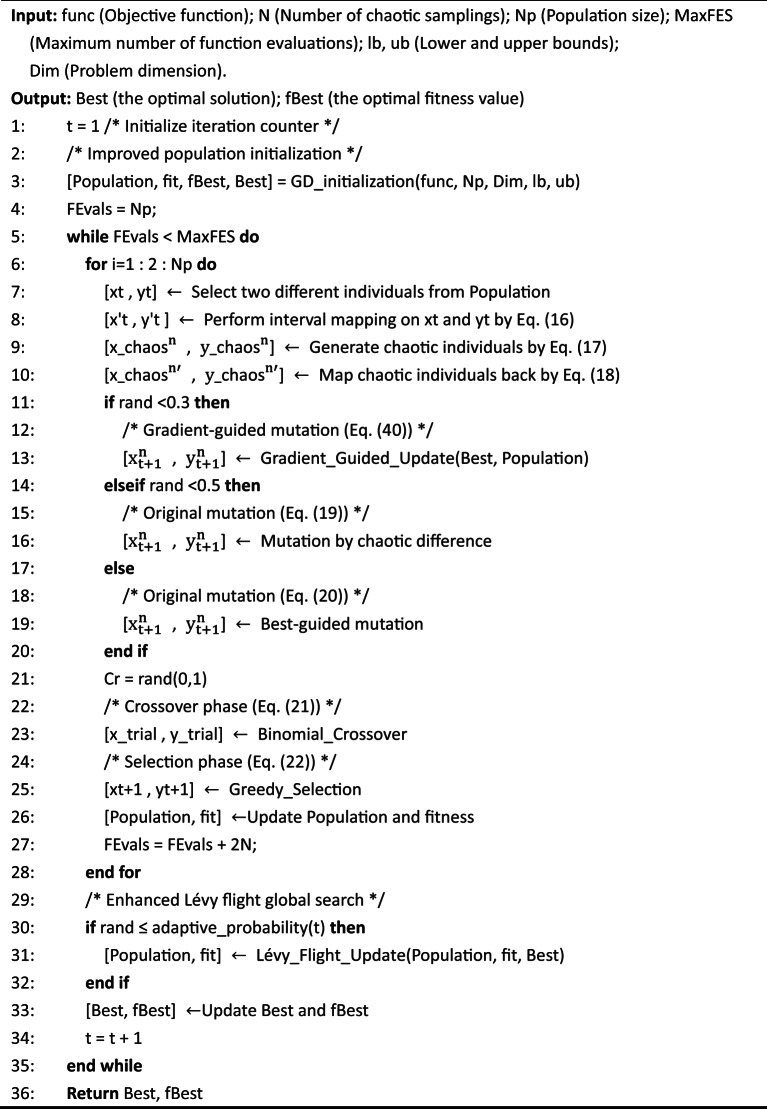
Pseudo-code of ICEO.

### Computational time complexity of ICEO

A good optimization algorithm should achieve strong performance on the problem; however, computational efficiency is also crucial, especially when the problem size and complexity increase. When applied practically, this algorithm delivers a good quality solution along with running time. Time complexity gives you how much computational cost goes up when you change the population and problem dimensions, which helps give some grounds theoretically when scaling up or real time.

With respect to the regular CEO algorithm it has an O(N×D) computation cost because of its control parameter as well as position vector computation part, here N indicates the size of our population whereas D represents the issue’s dimensions. In the start of creation we have O(N×D), for formation of the set of individuals as well as assessment of first fitness value considering that each function of evaluation needs amount of D. In terms of the iterative step each individual was updated every single round. Since chaotic mapping, mutation, crossover, and fitness evaluation are all implemented via dimension-wise vector operations, the per-iteration complexity is O(N×D). Over T iterations, the total computational complexity becomes O(T×N×D). Therefore, the overall time complexity of CEO is O(T×N×D).

The proposed ICEO algorithm introduces additional mechanisms, including multi-scale gradient memory, adaptive learning rate adjustment, and global search based on Lévy flight. Although these improvements increase the fixed computational overhead due to the additional vector-based operations, they do not change the asymptotic order of complexity. Consequently, the overall computational complexity of ICEO remains O(T×N×D).

## Algorithm performance testing and analysis

All experiments in this paper were conducted based on the following hardware and software environment: an Intel(R) Core(TM) Ultra 5 125 H processor, 32 GB of RAM, and the Windows 10 operating system. Both the performance testing and the UAV 3D path planning simulation was conducted on the MATLAB R2023a platform.

The experimental test datasets include standard benchmark functions from CEC2017^39^, CEC2020^40^, and CEC2022^41^. The comparative algorithms include classic metaheuristic algorithms such as grey wolf optimizer (GWO)^42^, Whale optimization algorithm (WOA)^43^, and particle swarm optimization (PSO)^44^, as well as recently proposed algorithms, including spider wasp optimization (SWO)^45^, dream optimization algorithm (DOA)^46^, polar lights optimizer (PLO)^47^, educational competition optimizer (ECO)^48^, and chaotic evolution optimization (CEO).

### Performance verification of ICEO

To fully assess the performance of the proposed ICEO algorithm on various kinds of complicated optimization issues, this section uses three widely recognized international benchmark test function sets: CEC2017, CEC2020, and CEC2022.

CEC2017 has 30 continuous optimization functions: 3 unimodal functions (F1-F3), 6 multimodal functions (F4-F10), and 21 hybrid and composite functions (F11-F30). Unimodal function is used to test the local search accuracy of the algorithm, and multimodal function is used to test the global optimization ability of the algorithm. Hybrid and composite functions have variable coupling and nonlinearity, making the problem more difficult and testing the stability and global optimization capability of the algorithm at various difficulty levels.

The CEC2020 test suite has 10 functions. In comparison to CEC2017, it has added rotational coupling, nonlinear transformations, and shift operations, making the functions have a greater degree of asymmetry and nonlinearity, greatly increasing the difficulty of optimization.

The CEC2022 test suite consists of 12 functions, in which high-dimensional nonlinear coupling and non-separability are further strengthened, and variable dependencies are more pronounced.

**Fig. 7:**
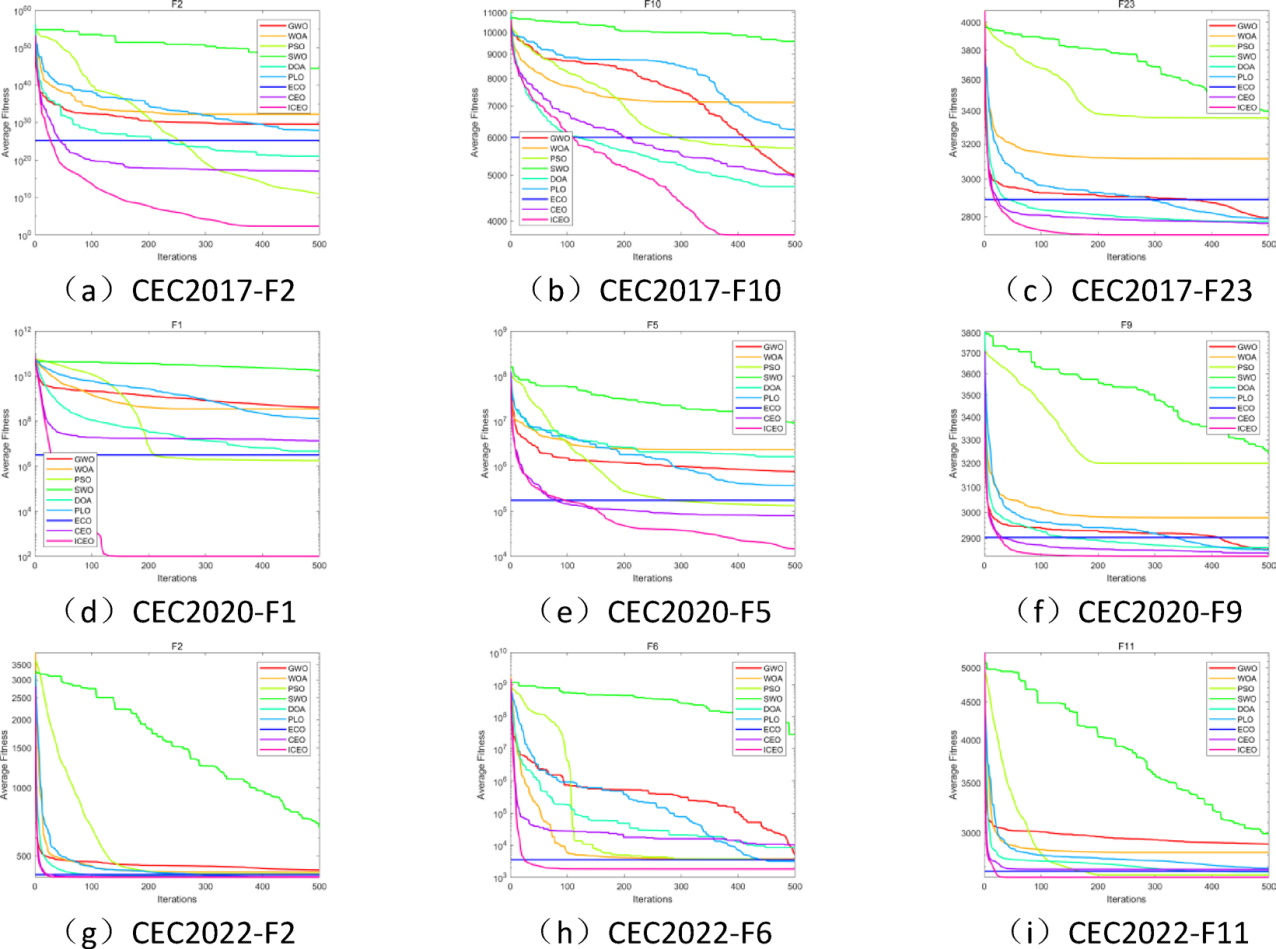
Convergence curves of representative CEC benchmark functions.

**Fig. 8:**
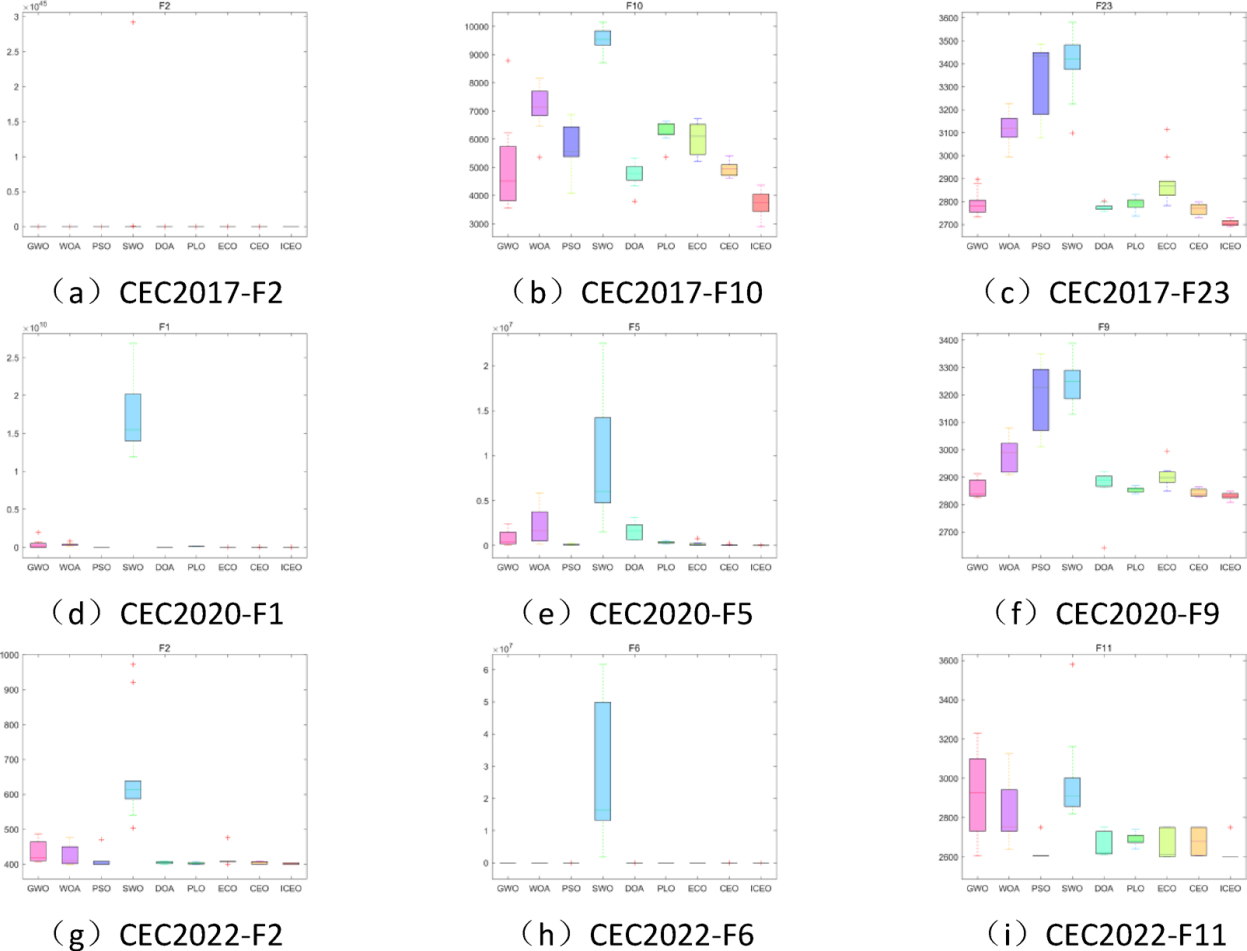
Box Plots of partial experimental results on CEC benchmark suites.

**Fig. 9:**
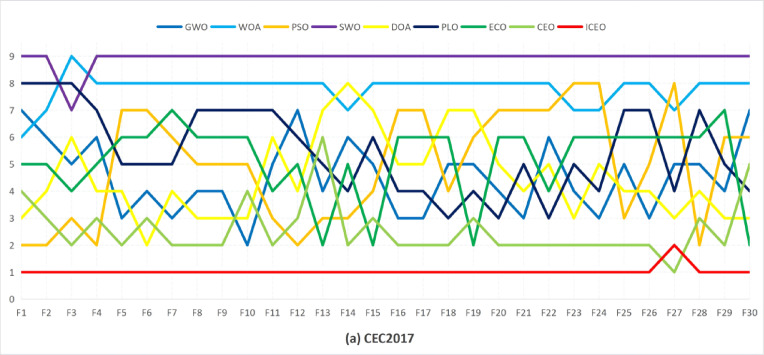

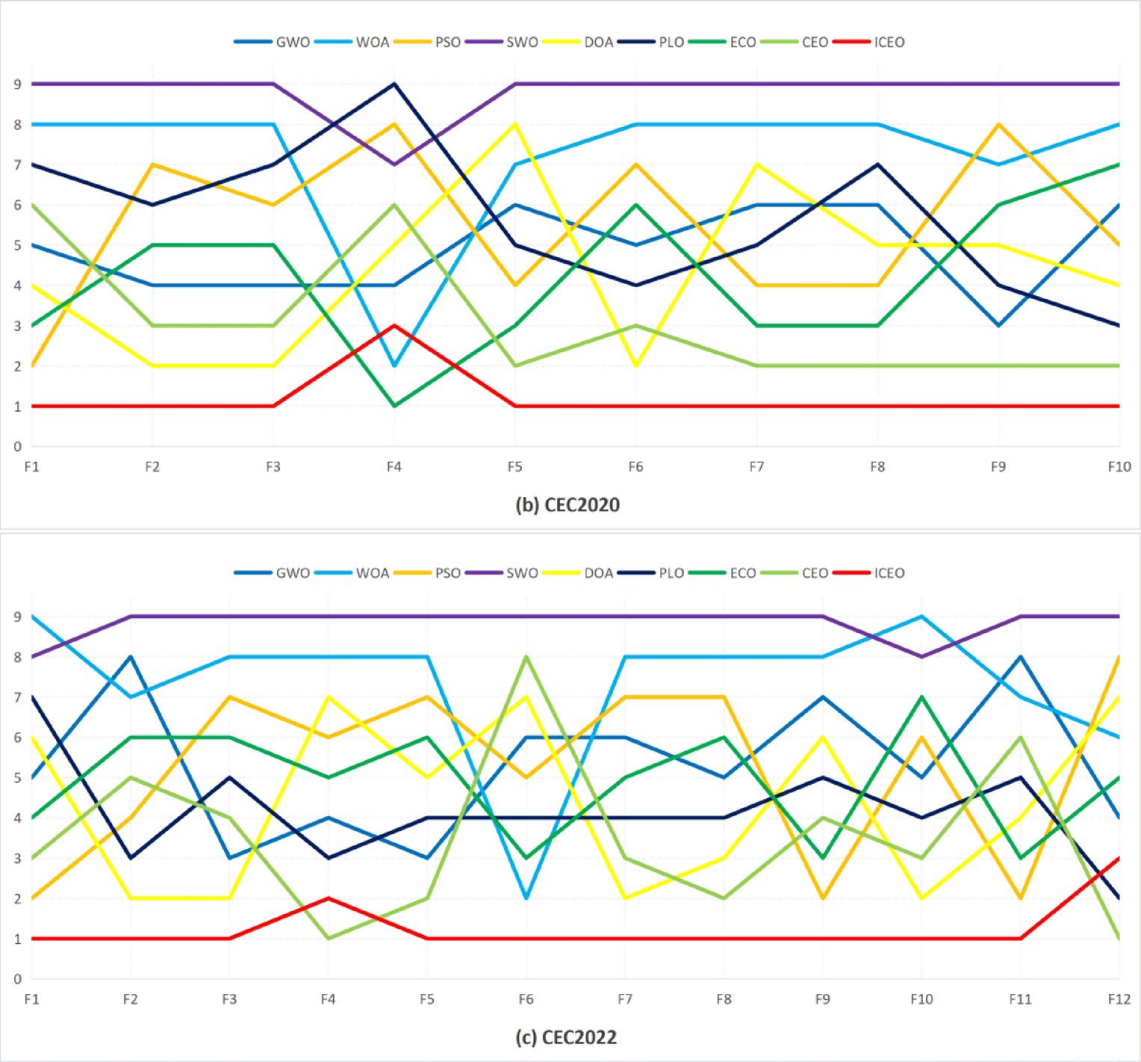
Friedman ranking results of the three test sets.

For all algorithms, the population size was fixed at 50, and the maximum number of iterations was 500. To decrease randomness, each algorithm was executed 10 times on each test function, and the average best value (ave) and standard deviation (std) were recorded. A subset of representative benchmark functions was selected for experimental evaluation, covering unimodal functions, simple multimodal functions, hybrid functions, and composition functions. Specifically, the selected functions include F2 (No.1), F10 (No.2), and F23 (No.3) from CEC 2017; F1 (No.4), F5 (No.5), and F9 (No.6) from CEC 2020; as well as F2 (No.7), F6 (No.8), and F11 (No.9) from CEC 2022. The experimental results are given in Table 1, and the corresponding convergence curves are shown in Fig. 7. and the box plots in Fig. 8. The Friedman rankings on the three test sets are presented in Fig. 9.

**Table 1:** Partial experimental results on CEC benchmark suites.

No.	Metric	GWO	WOA	PSO		SWO	DOA	PLO		ECO	CEO	ICEO	
1	ave	3.78E + 29	1.65E + 32	9.83E + 10		2.92E + 44	1.12E + 21	7.65E + 27		1.75E + 25	1.11E + 17	**2.00E + 02**	
	std	1.17E + 30	3.41E + 32	2.95E + 11		9.23E + 44	2.32E + 21	7.72E + 27		5.44E + 25	3.00E + 17	**2.35E-05**	
2	ave	5.01E + 03	7.12E + 03	5.70E + 03		9.57E + 03	4.73E + 03	6.24E + 03		6.01E + 03	4.96E + 03	**3.74E + 03**	
	std	1.58E + 03	7.97E + 02	8.14E + 02		4.28E + 02	4.36E + 02	3.75E + 02		5.77E + 02	**2.85E + 02**	4.66E + 02	
3	ave	2.79E + 03	3.11E + 03	3.36E + 03		3.40E + 03	2.78E + 03	2.79E + 03		2.89E + 03	2.77E + 03	**2.71E + 03**	
	std	5.50E + 01	7.26E + 01	1.50E + 02		1.40E + 02	1.43E + 01	3.10E + 01		9.74E + 01	2.50E + 01	**1.42E + 01**	
4	ave	4.10E + 08	3.46E + 08	1.76E + 06		1.75E + 10	4.56E + 06	1.30E + 08		3.11E + 06	1.35E + 07	**1.00E + 02**	
	std	6.15E + 08	1.79E + 08	2.27E + 05		5.36E + 09	1.88E + 06	1.79E + 07		2.35E + 06	4.39E + 06	**6.70E-15**	
5	ave	7.53E + 05	2.32E + 06	1.33E + 05		9.03E + 06	1.62E + 06	3.68E + 05		1.76E + 05	7.91E + 04	**1.47E + 04**	
	std	8.07E + 05	1.93E + 06	8.13E + 04		6.90E + 06	8.59E + 05	1.16E + 05		2.38E + 05	7.44E + 04	**1.92E + 04**	
6	ave	2.86E + 03	2.98E + 03	3.20E + 03		3.25E + 03	2.86E + 03	2.86E + 03		2.90E + 03	2.84E + 03	**2.83E + 03**	
	std	3.41E + 01	5.78E + 01	1.22E + 02		7.92E + 01	8.02E + 01	**1.02E + 01**		3.96E + 01	1.43E + 01	1.21E + 01	
7	ave	4.33E + 02	4.25E + 02	4.17E + 02		6.64E + 02	4.05E + 02	4.03E + 02		4.14E + 02	4.05E + 02	**4.02E + 02**	
	std	3.01E + 01	3.00E + 01	2.85E + 01		1.55E + 02	3.13E + 00	2.19E + 00		2.22E + 01	3.43E + 00	**2.06E + 00**	
8	ave	5.34E + 03	3.74E + 03	3.50E + 03		2.75E + 07	8.45E + 03	3.12E + 03		3.51E + 03	1.04E + 04	**1.80E + 03**	
	std	1.80E + 03	2.06E + 03	1.24E + 03		2.23E + 07	4.71E + 03	3.59E + 02		1.79E + 03	3.20E + 03	**1.99E + 00**	
9	ave	2.90E + 03	2.83E + 03	2.63E + 03		2.99E + 03	2.66E + 03	2.69E + 03		2.66E + 03	2.68E + 03	**2.62E + 03**	
	std	2.17E + 02	1.68E + 02	6.13E + 01		2.28E + 02	6.01E + 01	**3.47E + 01**		7.56E + 01	7.52E + 01	4.76E + 01	

In the 30-dimensional test functions of CEC2017, ICEO demonstrated clear advantages. Regarding the unimodal function F2, ICEO obtained a mean optimal solution which exceeded both the initial CEO and alternative benchmark methods by multiple orders of magnitude, featuring a standard deviation considerably smaller than those of the rivals. On the multimodal function F10, ICEO reached the minimal average optimal result. For the hybrid function F23, ICEO not only achieved the superior average performance but also kept a standard deviation substantially smaller than the various competing techniques.

In the 20-dimensional test functions of CEC2020, the performance advantage of ICEO was further demonstrated. For the 3 functions in the table, ICEO had the highest average value and the lowest standard deviation.

For the 10-dimensional test function of CEC2022, the gap between different algorithms on some unimodal functions was smaller than before, but ICEO was still leading overall.

The Friedman test is a non-parametric analytical technique employed to evaluate several algorithms across diverse benchmark problems without requiring the data to adhere to a Gaussian distribution. Within this procedure, different algorithms are ranked based on their mean optimal objective values for every benchmark function, where the rank 1 is given to the most effective algorithm. Subsequently, the average ranking of each algorithm across all evaluation functions will be calculated. Then, statistical analysis will be conducted to determine whether the differences between these average rankings are statistically significant. The smaller the average ranking, the better the general performance and the more stable the stability of the specific algorithm in various tasks.

Figure 9 presents the Friedman ranking results for each evaluation algorithm against three standard benchmark test suites (CEC2017, CEC2020, and CEC2022). Based on these findings, it is clearly observable that the proposed ICEO framework consistently maintains the highest average ranking across all three datasets, thereby confirming the improvement in its optimization performance and stability. Compared to classic algorithms such as GWO, WOA, and PSO, as well as recent meta-heuristic methods including SWO, DOA, PLO, ECO, and CEO, ICEO demonstrates a significant advantage in overall performance.

Based on the empirical results of these three standard test suites, it is clearly evident that ICEO demonstrates higher efficiency in continuous optimization challenges across various dimensions and categories. Compared with other methods, it achieves faster convergence speed, better optimization results, and higher stability. More intuitively, through the evaluation of convergence curves and box plot analysis, It is obvious that the multi-strategy improvement proposed in this paper effectively strengthens the global exploration capability and suppresses premature convergence, thus improving the overall algorithm robustness.

### Ablation study

To evaluate the effectiveness of each proposed optimization measure, we conducted ablation analysis by sequentially removing specific elements from the optimization algorithm. In this study, the original CEO method served as the reference, while ICEO represented the complete version that integrated all the introduced improvement techniques. Based on this framework, we constructed three ablation variants, sequentially removing each key component, namely ICEO1 (without the Good Point Set initialization), ICEO2 (without the multi-scale gradient guidance strategy based on Adam optimization), and ICEO3 (without the enhanced Lévy flight strategy). This experimental design helps to assess the contribution of each component to the overall performance of the proposed method, thereby enabling a clear quantification of the contribution of each component to the final performance.

An ablation study was conducted using the CEC2017 benchmark test suite, where each objective function was assigned to a 30-dimensional space. Each algorithm maintained a population size of 50 and a maximum number of iterations of 500. To reduce the influence of randomness, each optimizer ran 10 trials independently each time the function was evaluated. During the entire experiment, the fitness indicators for each iteration were recorded. Subsequently, the average optimal performance (ave), standard deviation (std), Friedman ranking, and Wilcoxon rank sum test results for each method on each function were determined. The comprehensive results are presented in Tables 2, 3 and 4.

The Wilcoxon rank sum test method is a non-parametric evaluation approach designed to determine whether two different groups come from the same distribution. Under this framework, the output results of the two methods will be combined and arranged in an ascending sequence. Then, rank values will be assigned to each technique, and based on this, the position of each data set will be calculated. Based on whether there is a significant difference in these two rank values, a statistical indicator for testing is constructed. The null hypothesis assumes that the two independent algorithms have the same efficiency level. If the obtained p-value is lower than the pre-set significance threshold (usually 0.05), it indicates that the difference in efficiency is statistically significant.

**Table 2:** Experimental results of ablation study on CEC2017 suite.

No.	Metric	CEO	ICEO1	ICEO2	ICEO3	ICEO
F1	ave	4.30E + 07	4.44E + 05	4.31E + 05	2.84E + 04	**1.00E + 02**
	std	1.42E + 07	1.65E + 05	1.51E + 05	1.21E + 04	**7.68E-07**
F2	ave	2.21E + 16	2.97E + 04	2.22E + 03	2.31E + 04	**2.05E + 02**
	std	3.71E + 16	4.84E + 04	4.00E + 03	6.46E + 04	**1.71E + 01**
F3	ave	5.32E + 03	2.84E + 03	1.70E + 03	4.54E + 02	**3.20E + 02**
	std	2.02E + 03	1.20E + 03	9.29E + 02	1.79E + 02	**2.72E + 01**
F4	ave	5.12E + 02	4.86E + 02	5.01E + 02	4.98E + 02	**4.36E + 02**
	std	**1.57E + 01**	3.51E + 01	2.10E + 01	1.72E + 01	3.38E + 01
F5	ave	5.85E + 02	5.55E + 02	5.56E + 02	5.55E + 02	**5.52E + 02**
	std	2.17E + 01	1.03E + 01	**1.01E + 01**	1.58E + 01	1.63E + 01
F6	ave	6.04E + 02	6.00E + 02	6.00E + 02	6.00E + 02	**6.00E + 02**
	std	8.72E-01	6.22E-02	5.88E-02	4.18E-02	**1.05E-10**
F7	ave	8.74E + 02	7.83E + 02	7.91E + 02	7.86E + 02	**7.80E + 02**
	std	1.24E + 01	1.42E + 01	2.02E + 01	1.24E + 01	**1.04E + 01**
F8	ave	8.81E + 02	8.56E + 02	8.55E + 02	8.53E + 02	**8.48E + 02**
	std	2.75E + 01	1.34E + 01	1.66E + 01	1.51E + 01	**1.05E + 01**
F9	ave	9.56E + 02	9.15E + 02	9.15E + 02	9.11E + 02	**9.08E + 02**
	std	2.66E + 01	1.03E + 01	1.93E + 01	8.23E + 00	**7.49E + 00**
F10	ave	5.05E + 03	4.04E + 03	3.91E + 03	3.30E + 03	**2.99E + 03**
	std	3.62E + 02	3.74E + 02	**3.59E + 02**	5.94E + 02	5.03E + 02
F11	ave	1.22E + 03	1.15E + 03	**1.14E + 03**	1.15E + 03	1.15E + 03
	std	3.26E + 01	3.10E + 01	**2.05E + 01**	2.47E + 01	2.54E + 01
F12	ave	7.72E + 06	6.10E + 05	4.77E + 05	5.24E + 05	**3.04E + 04**
	std	3.49E + 06	3.70E + 05	1.95E + 05	2.50E + 05	**1.44E + 04**
F13	ave	1.52E + 06	5.73E + 04	4.10E + 04	2.16E + 04	**3.14E + 03**
	std	6.29E + 05	2.38E + 04	9.91E + 03	9.93E + 03	**3.37E + 03**
F14	ave	8.69E + 03	4.34E + 03	3.39E + 03	2.82E + 03	**2.24E + 03**
	std	7.43E + 03	2.28E + 03	2.01E + 03	1.44E + 03	**9.47E + 02**
F15	ave	6.63E + 04	4.83E + 03	6.81E + 03	4.26E + 03	**2.32E + 03**
	std	1.48E + 04	1.63E + 03	3.86E + 03	**1.34E + 03**	1.54E + 03
F16	ave	2.29E + 03	2.10E + 03	2.08E + 03	2.11E + 03	**2.06E + 03**
	std	2.25E + 02	**1.18E + 02**	2.61E + 02	1.79E + 02	1.67E + 02
F17	ave	1.94E + 03	**1.77E + 03**	1.83E + 03	1.84E + 03	1.80E + 03
	std	8.66E + 01	**3.80E + 01**	5.48E + 01	8.67E + 01	8.01E + 01
F18	ave	2.07E + 05	1.94E + 05	1.39E + 05	1.45E + 05	**7.58E + 04**
	std	1.08E + 05	1.14E + 05	1.05E + 05	7.30E + 04	**4.58E + 04**
F19	ave	9.52E + 04	7.13E + 03	8.38E + 03	6.63E + 03	**2.25E + 03**
	std	4.33E + 04	5.53E + 03	3.75E + 03	6.57E + 03	**9.09E + 02**
F20	ave	2.23E + 03	2.22E + 03	2.14E + 03	2.14E + 03	**2.12E + 03**
	std	9.47E + 01	1.03E + 02	8.19E + 01	8.82E + 01	**7.01E + 01**
F21	ave	2.40E + 03	2.35E + 03	**2.34E + 03**	2.35E + 03	2.36E + 03
	std	2.32E + 01	1.28E + 01	**1.23E + 01**	1.32E + 01	1.70E + 01
F22	ave	2.33E + 03	2.55E + 03	2.31E + 03	2.30E + 03	**2.30E + 03**
	std	3.41E + 00	7.50E + 02	1.32E + 00	**9.92E-01**	1.25E + 00
F23	ave	2.77E + 03	2.71E + 03	2.71E + 03	2.71E + 03	**2.70E + 03**
	std	2.62E + 01	2.00E + 01	1.69E + 01	1.51E + 01	**1.24E + 01**
F24	ave	2.91E + 03	2.89E + 03	**2.88E + 03**	2.88E + 03	2.88E + 03
	std	2.54E + 01	2.96E + 01	**1.55E + 01**	1.60E + 01	1.73E + 01
F25	ave	2.89E + 03	2.89E + 03	2.89E + 03	2.89E + 03	**2.89E + 03**
	std	8.62E + 00	3.42E + 00	**1.31E + 00**	5.03E + 00	1.75E + 00
F26	ave	4.30E + 03	4.25E + 03	4.38E + 03	4.34E + 03	**3.96E + 03**
	std	7.45E + 02	2.32E + 02	6.04E + 02	2.48E + 02	**2.17E + 02**
F27	ave	3.22E + 03	3.22E + 03	3.22E + 03	3.22E + 03	**3.21E + 03**
	std	1.49E + 01	1.06E + 01	9.56E + 00	7.38E + 00	**6.22E + 00**
F28	ave	3.25E + 03	3.21E + 03	3.20E + 03	3.22E + 03	**3.14E + 03**
	std	1.70E + 01	1.48E + 01	4.90E + 01	2.01E + 01	**7.93E + 00**
F29	ave	3.62E + 03	3.43E + 03	3.44E + 03	3.44E + 03	**3.41E + 03**
	std	9.21E + 01	6.47E + 01	6.53E + 01	8.28E + 01	**5.26E + 01**
F30	ave	1.45E + 06	4.08E + 04	3.77E + 04	2.52E + 04	**7.27E + 03**
	std	3.13E + 05	1.35E + 04	1.41E + 04	7.40E + 03	**2.29E + 03**

In the CEC2017 benchmark test, it is clearly observable that the proposed ICEO achieved the best overall performance among all the compared variants. Its average value was always the lowest on most of the test functions, while the original CEO performed the worst. This indicates that the proposed improvement measures are effective. The three ablated variants (ICEO1, ICEO2, and ICEO3) exhibit different performance characteristics across various types of functions, reflecting the distinct contributions of each module.

For unimodal functions (F1–F3), all three variants significantly outperform the baseline CEO. ICEO2 generally has the smallest average so its more accurate when optimized. ICEO3 shows lower variability in certain functions, indicating a more stable convergence process. The performance of ICEO1 is relatively moderate, suggesting that although the initialization strategy improves performance, its contribution to the overall performance is relatively limited and smaller compared to the other two mechanisms.

For multimodal functions (F4-F10), each of them has its own distinct advantages. Compared with other algorithms, ICEO3 can achieve lower standard deviations in some functions, thus being more robust and better able to avoid local optimal solutions. ICEO2 can achieve comparable or lower average values on some functions, indicating that it is very effective in guiding the search direction. ICEO1 has been continuously improving, but the improvement is very small, mainly in functions with more peaks and valleys.

For hybrid functions (F11-F20), ICEO2 demonstrates superior optimization accuracy across multiple functions, although its standard deviation is relatively larger in some cases, suggesting a decrease in stability. In contrast, ICEO3 has better stability with smaller fluctuations, while ICEO1’s performance lies between the other two variants, but its competitiveness is slightly inferior to the other two variations.

For composition functions (F21-F30), there are significant differences among these three variant versions. The average value of ICEO2 is lower on some functions, indicating that it has a stronger search ability; while ICEO3 shows better stability with a smaller standard deviation. ICEO1 performs relatively well on simpler combined functions, but shows limited improvement on more complex functions.

Overall, the results of the ablation experiments indicate that each improved component makes a distinct contribution to the performance of the algorithm. The multi-scale gradient guidance strategy (ICEO2) mainly enhances the accuracy of the solution, the enhanced Lévy flight strategy (ICEO3) significantly improves stability and global exploration capabilities, while the Good Point Set initialization (ICEO1) provides supplementary improvements in population diversity. However, no portion on its own would get far. And so all put together is having the highest possible balance of preciseness and strength, and thus it improves on its whole ability to find answers as well as its speed.

Friedman ranking results show that ICEO gets an average score of 1.4 on all functions, much better than CEO (4.9), ICEO1 (2.4), ICEO2 (2.9), and ICEO3 (3.4), which also proves that the multiple strategy combination has a general advantage.

Wilcoxon rank sum test results indicate that ICEO is different from the rest of the versions for most functions (*p* < 0.05), which shows that the improvement made by each strategy can be considered as a statistically meaningful improvement to the algorithm’s performance.

**Table 3:** Friedman test rankings of ICEO and its ablation versions.

No.	CEO	ICEO1	ICEO2	ICEO3	ICEO
F1	5	2	3	4	1
F2	5	2	4	3	1
F3	5	2	3	4	1
F4	5	2	3	4	1
F5	5	3	1	4	2
F6	5	2	3	4	1
F7	5	4	2	3	1
F8	5	4	3	2	1
F9	5	3	1	4	2
F10	5	3	2	4	1
F11	5	4	1	3	2
F12	5	2	4	3	1
F13	5	1	3	4	2
F14	5	1	4	3	2
F15	5	2	3	4	1
F16	5	3	4	2	1
F17	5	1	4	3	2
F18	5	1	3	4	2
F19	5	1	3	4	2
F20	5	4	2	3	1
F21	5	1	4	3	2
F22	5	2	3	4	1
F23	5	2	4	3	1
F24	5	2	1	4	3
F25	5	4	1	3	2
F26	5	3	2	4	1
F27	3	4	5	2	1
F28	5	2	4	3	1
F29	5	2	3	4	1
F30	5	2	3	4	1
Mean rank	4.9	2.4	2.9	3.4	1.4

**Table 4:** Wilcoxon rank-sum test results.

No.	CEO	ICEO1	ICEO2	ICEO3	ICEO
F1	1.83E-04	1.83E-04	1.83E-04	1.83E-04	NaN
F2	1.83E-04	2.46E-04	3.30E-04	4.40E-04	NaN
F3	1.83E-04	1.83E-04	1.83E-04	1.70E-03	NaN
F4	1.83E-04	3.60E-03	7.69E-04	1.83E-04	NaN
F5	1.00E-03	4.73E-01	7.91E-01	6.78E-01	NaN
F6	1.83E-04	1.83E-04	1.83E-04	1.83E-04	NaN
F7	1.83E-04	6.23E-01	6.78E-01	2.12E-01	NaN
F8	3.12E-02	9.70E-01	2.73E-01	1.00E + 00	NaN
F9	7.69E-04	1.21E-01	5.71E-01	1.86E-01	NaN
F10	1.83E-04	4.40E-04	1.00E-03	4.27E-01	NaN
F11	5.83E-04	9.70E-01	4.73E-01	9.70E-01	NaN
F12	1.83E-04	1.83E-04	1.83E-04	1.83E-04	NaN
F13	1.83E-04	1.83E-04	1.83E-04	3.30E-04	NaN
F14	2.46E-04	9.10E-03	1.86E-01	2.41E-01	NaN
F15	1.83E-04	2.20E-03	1.30E-03	7.30E-03	NaN
F16	2.11E-02	9.10E-01	3.85E-01	8.50E-01	NaN
F17	3.60E-03	7.91E-01	7.57E-02	3.08E-01	NaN
F18	2.20E-03	1.13E-02	2.12E-01	2.57E-02	NaN
F19	1.83E-04	4.40E-04	3.30E-04	1.00E-03	NaN
F20	4.52E-02	7.57E-02	5.71E-01	9.10E-01	NaN
F21	1.13E-02	1.41E-01	2.57E-02	7.57E-02	NaN
F22	1.59E-04	1.59E-04	1.59E-04	1.32E-02	NaN
F23	5.83E-04	1.86E-01	7.34E-01	4.73E-01	NaN
F24	1.41E-01	8.90E-02	8.90E-02	1.21E-01	NaN
F25	1.73E-02	5.71E-01	7.34E-01	6.78E-01	NaN
F26	2.41E-01	2.12E-01	1.41E-01	9.70E-01	NaN
F27	3.45E-01	9.70E-01	1.00E + 00	7.34E-01	NaN
F28	1.83E-04	5.80E-03	5.39E-02	4.60E-03	NaN
F29	2.46E-04	5.21E-01	2.41E-01	4.73E-01	NaN
F30	1.83E-04	1.83E-04	1.83E-04	1.83E-04	NaN

## Application of ICEO in 3D UAV path planning

Regarding the UAV 3D path planning experiment, to guarantee the equity and consistency of the evaluation, we established the swarm dimension at 50 and the highest iteration count to 200 across every trial. Each algorithm was executed autonomously 10 iterations, and the final results were analyzed via statistical metrics. Variables not specified within the original problem were configured to a standard level during this process as illustrated hereafter.

The UAV flight path was defined using 10 control points. The UAV’s minimum and maximum flight altitudes were set to $\:{z}_{min}=100$ and $\:{z}_{max}=200$, respectively; the UAV’s equivalent radius was set to $\:{r}_{d}=10$, with a safety buffer distance $\:S=2{r}_{d}$; the maximum allowable turning angle threshold was set to $\:{\theta\:}_{max}=45^\circ\:$, and the maximum allowable change in climb angle was set to $\:{\phi\:}_{max}=45^\circ\:$.

### Simulation scenario design

Experimental terrain environment was created according to the actual terrain data of Australia, and a 3D elevation model was formed. To simulate the distribution of threats under various operating or monitoring conditions, three groups of threat zones with different spatial arrangements were designed. The exact spatial coordinates and related radius parameters for these three sets of threat zones are shown in Table 5.

**Table 5:** Configuration of threat zones for the three simulation scenarios.

Scenario 1						
Center	(400,500,200)	(600,200,200)	(500,350,200)	(350,200,200)	(700,550,200)	(650,750,150)
Radius	50	40	50	40	40	50

Scenario 2						
Center	(280,360,190)	(520,180,200)	(740,320,180)	(610,620,200)	(420,750,170)	(300,580,160)
Radius	45	50	40	50	45	40

Scenario 3						
Center	(300,300,180)	(450,320,200)	(600,300,180)	(650,500,200)	(500,500,180)	(350,480,160)
Radius	45	50	45	50	40	45

**Fig. 10:**
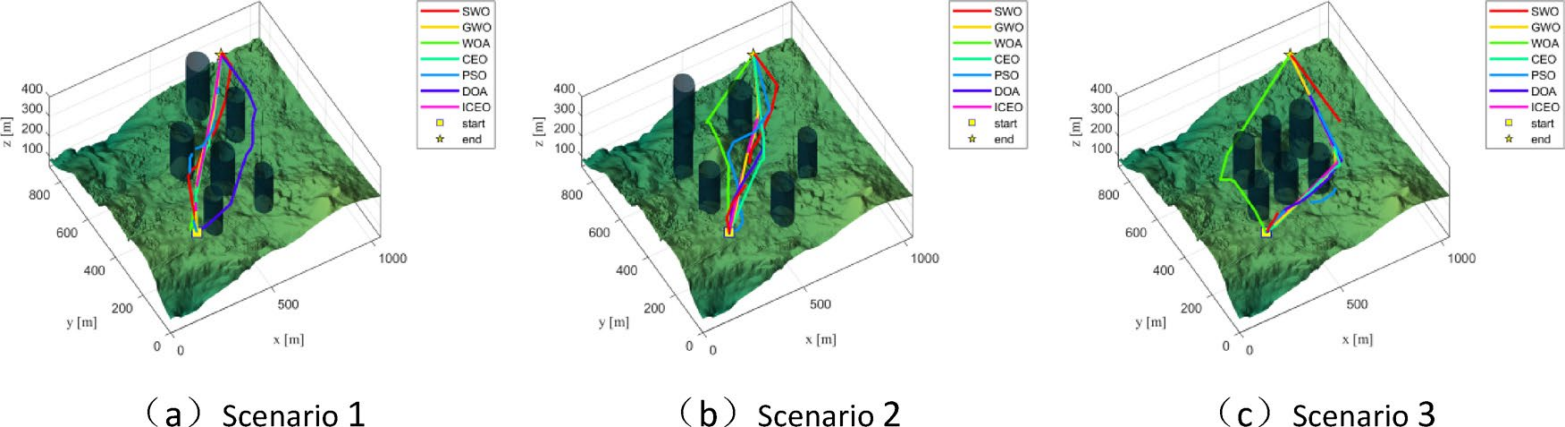
3D views of the flight paths generated by each algorithm.

**Fig. 11:**
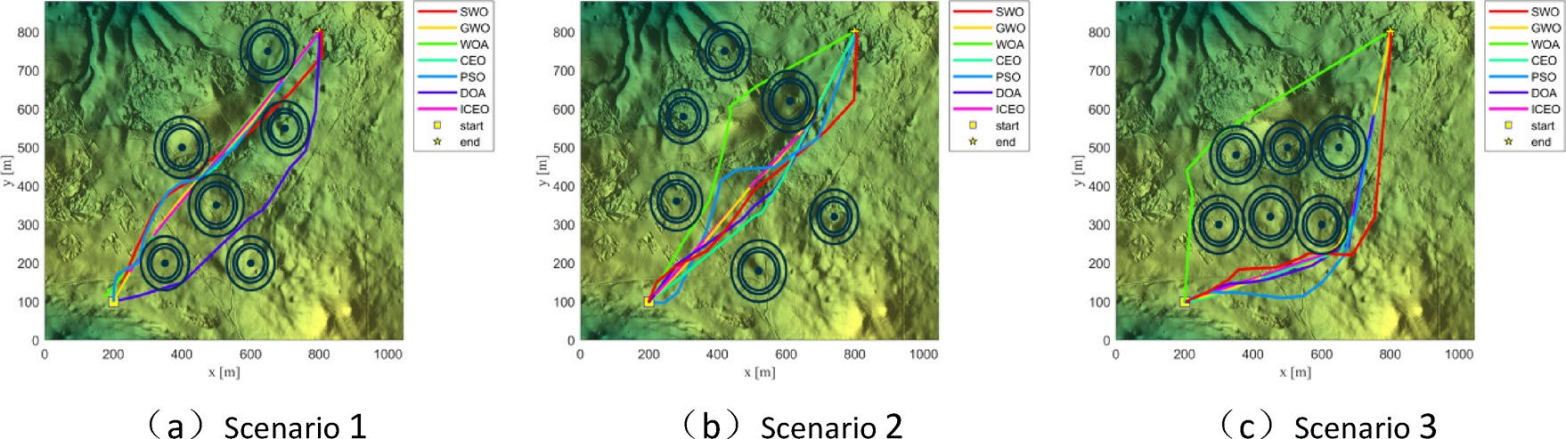
Top views of the flight paths generated by each algorithm.

**Fig. 12:**
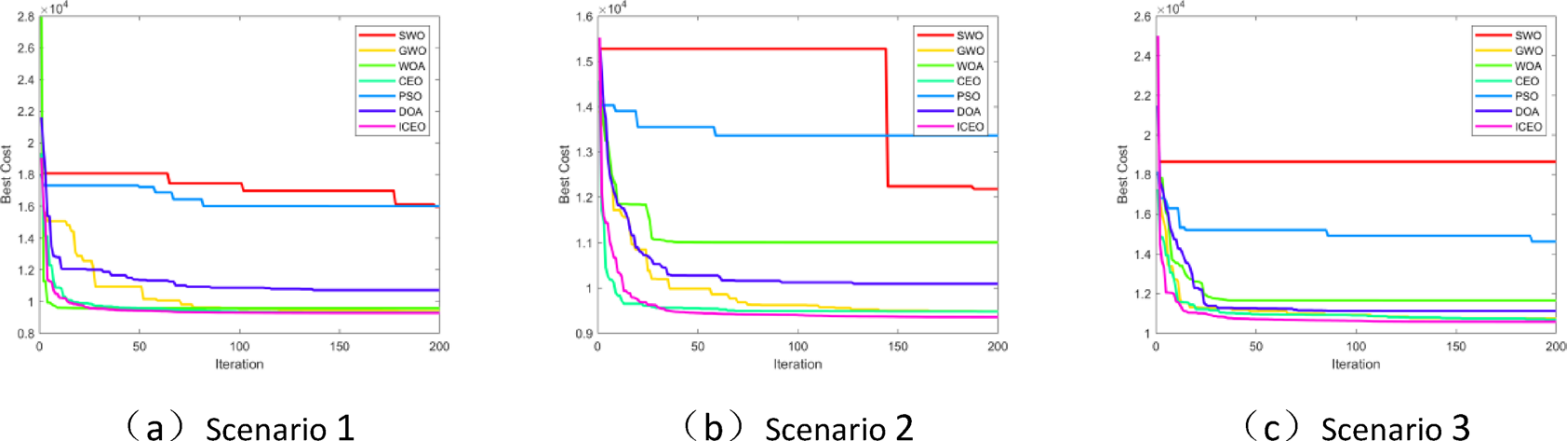
Convergence curves of each algorithm.

In the three typical cases mentioned above, all the algorithms could produce flight paths that meet the basic feasibility conditions. But there was quite a difference when it came to how good the paths were, how steady they were, and how well they were made better. The related statistical data are listed in Tables 6, 7 and 8, and the path visualization and convergence characteristics can be seen in Figs.10, 11 and 12.

**Table 6:** Statistical results for scenario 1.

Algorithm	Best cost	Worst cost	Mean cost	Std
SWO	14050.14324	17741.17121	15370.94655	1103.716572
GWO	9355.694834	9572.262434	9433.92983	66.21863326
WOA	9584.422558	10101.91714	9649.686377	161.2091018
CEO	9312.527201	9591.604986	9386.952103	82.79743506
PSO	12718.10114	15666.35575	14473.58085	1173.071344
DOA	9567.973837	10930.85481	10237.74708	468.5590737
ICEO	**9280.250938**	**9306.827491**	**9292.788567**	**9.656061802**

**Table 7:** Statistical results for scenario 2.

Algorithm	Best cost	Worst cost	Mean cost	Std
SWO	11819.84245	13874.55646	12920.94932	624.2132432
GWO	9410.922366	9510.601716	9444.348762	32.85423259
WOA	10888.10267	14154.18743	12219.93551	973.7910384
CEO	9374.443656	9532.663166	9419.30758	54.08608257
PSO	12138.5511	13819.82148	13145.01434	549.3602227
DOA	9687.639477	10120.63895	9833.716367	139.4654104
ICEO	**9356.244203**	**9379.308495**	**9371.399401**	**8.652200008**

**Table 8:** Statistical results for scenario 3.

Algorithm	Best cost	Worst cost	Mean cost	Std
SWO	13664.44564	18641.39321	16089.79755	1608.207334
GWO	10670.37495	10892.49253	10764.55552	59.63148401
WOA	11314.83258	14957.33846	13117.82478	1404.77757
CEO	10645.62337	10751.62074	10692.88676	37.92571163
PSO	13564.47066	16455.46343	14939.84725	763.9571204
DOA	10968.27858	11636.76235	11217.69971	196.6469331
ICEO	**10591.12834**	**10626.15906**	**10604.10547**	**10.45553457**

### Analysis of path planning results

From the statistics of the three experiments, it can be seen that ICEO has obvious advantages in some important indicators. Regardless of which one is considered, the shortest path length, the longest path length, or the average path length, or the standard deviation, ICEO performs the best in all cases. Corresponding data is marked in bold in the table.

Path visualization results show that SWO, PSO, and WOA generate paths with long detours, some trajectories are near threat zones and there are a lot of sharp turns. GWO and CEO have relatively stable routes that are mostly continuous and smooth, but they also have redundant sections and bigger detours in areas with lots of dangers. ICEO performs best for all three cases. It avoids danger zones well and keeps the route unbroken. In both the 3D and top views, it can be seen that ICEO can avoid these unnecessary detours with guaranteed safety so therefore our routes get smoother and cheaper for us compared to other algorithms.

In terms of convergence curves it can be seen here that each and every one of those algorithms manage quite well, in a very short interval at their start to be quite rapid at reducing the value for the objective function. But as more iterations happen, how well these kinds of algorithms get better at working changes too. During mid-late stages there is a significant slow down in convergence speed for SWO, WOA and PSO but GWO and CEO show near constant convergence though not totally precise in all the cases. While ICEO on the contrary has far swifter yet even pace convergence on all three occasions. The value of the objective quickly decreases from the initial part to the middle of iterations then remains almost stationary towards the end, without much variance, indicating great stability when optimizing and being strong and unchangeable.

## Conclusion

This paper proposes an improved chaotic evolution optimization (ICEO) algorithm for complex constrained 3D UAV path planning problems. The proposed method combines a good point set-based initialization strategy, a direction-aware multi-scale search mechanism, and an adaptive Lévy flight strategy to enhance the diversity, convergence stability and global exploration ability of the population during the optimization process.

The experimental results on the CEC benchmark test set show that the proposed method outperforms several representative meta-heuristic algorithms in terms of optimization performance and convergence stability. The statistical analysis based on Friedman ranking and Wilcoxon rank sum test further confirms the effectiveness of the proposed optimization strategy. Additionally, the ablation experiments demonstrate that the introduced mechanism collaboratively promotes exploration ability, refinement of solutions, and stability of optimization in different stages of the search process.

In the simulated 3D UAV path planning, the proposed method is capable of generating feasible, smooth and relatively efficient flight trajectories under multiple coupled constraints such as path safety, altitude limitations and trajectory smoothness. The results show that this method has potential application value in the constrained trajectory optimization problem in complex environments. Although the proposed method demonstrates promising optimization capability, several limitations still remain in the present work. The current study mainly focuses on simulation-based evaluation of constrained trajectory optimization performance in complex environments, where the experiments are designed to verify the effectiveness of the proposed algorithm under multiple coupled constraints, including path safety, trajectory smoothness, and altitude limitations.

The current experiments are limited to static environments and do not explicitly address issues such as dynamic obstacle avoidance, online re-planning, or environmental uncertainty. In actual robot navigation tasks, UAV typically operate in dynamic and partially observable environments, which include moving obstacles, sensor noise, communication delays, and unexpected task updates. Therefore, real-time response capabilities and adaptive re-planning abilities are of crucial importance.

Furthermore, due to the adoption of a population-based iterative optimization mechanism, the proposed method incurs higher computational costs compared to traditional graph-search and sampling-based planning methods. Therefore, planning methods such as A*, Dijkstra, PRM, and RRT, which are based on classic planning approaches, are still more suitable for real-time navigation and dynamic re-planning tasks in terms of computational efficiency and mature deployment capabilities. In contrast, the proposed ICEO algorithm is more applicable to offline optimization or advanced trajectory refinement problems involving complex nonlinear constraints and multiple coupled objectives.

Furthermore, the current research does not include hardware-level verification, airborne deployment analysis, or actual UAV flight experiments. Therefore, the practical applicability of the proposed framework in actual robotic systems still requires further study.

The future work will focus on further enhancing the applicability of the proposed framework in more realistic scenarios of UAV trajectory optimization. Possible research directions include combining ICEO with classical planning methods to construct a hierarchical planning framework; integrating prediction models and online re-planning mechanisms to adapt to dynamic environments; and verifying the proposed framework through hardware-in-the-loop simulations and actual UAV experiments. Extending the proposed method to multi-UAV cooperative trajectory optimization and distributed mission planning will also be considered in future studies.

## Data Availability

No data available for public disclosure, please contact the corresponding author if needed.
